# Low-density lipoprotein cholesterol lowering therapy for the secondary prevention of atherosclerotic cardiovascular disease

**DOI:** 10.21542/gcsp.2020.39

**Published:** 2020-12-31

**Authors:** Parth N. Patel, Robert P. Giugliano

**Affiliations:** 1 Department of Medicine, Brigham & Women’s Hospital, Harvard Medical School, Boston, MA, USA; 2 Division of Cardiovascular Medicine, Brigham & Women’s Hospital, Harvard Medical School, Boston, MA, USA

## Abstract

Atherosclerotic cardiovascular disease (ASCVD) is highly prevalent and a major contributor to morbidity and mortality worldwide. Elevated blood cholesterol is a key driver of risk for atherosclerotic events, and patients with established ASCVD comprise a specific high-risk population in which low-density lipoprotein cholesterol (LDL-C) lowering therapy is strongly endorsed by multiple guidelines. An increasing number of medications across several pharmacologic classes are available today in clinical practice. Therefore, guidance on the appropriate use of these interventions is necessary for cost-effective solutions to managing residual atherothrombotic risk. In this review we summarize the key evidence supporting LDL-C lowering as described in the most recent 2018 multi-society Blood Cholesterol Guidelines, and provide a framework for optimizing LDL-C lowering therapy in secondary prevention populations.

## Introduction

Cardiovascular disease remains the leading cause of morbidity and mortality in the world and is estimated to account for one third of deaths globally each year^1–3^. Atherosclerotic cardiovascular disease (ASCVD), the dominant form of cardiovascular disease worldwide, is a chronic disorder of lipid deposition, vascular inflammation, and plaque formation that progresses sub-clinically before manifesting as ischemic stroke, myocardial infarction (MI), and peripheral limb ischemia. ASCVD events can be dramatic, at times leading to sudden and significant detriments to quality of life^4,5^. Fortunately, scientific advances have enhanced our understanding of the pathogenesis of ASCVD and have identified multiple risk factors responsible for its initiation and development over time. Clinical management of these risk factors has successfully decreased the burden of ASCVD over the last few decades^6^.

Elevated blood cholesterol is one of the primary causal risk factors for the development of ASCVD. Studies have shown that even modestly elevated levels of blood cholesterol increase rates of major adverse cardiac events^7–9^, and multiple guidelines support the use of cholesterol-lowering interventions in populations at elevated cardiovascular risk. Patients with a history of prior stroke, ischemic heart disease, or peripheral arterial disease comprise a specific population in which lipid-lowering therapy is guideline supported for secondary prevention in all patients^10^. In this review we summarize the key evidence supporting the most recent 2018 multi-society Blood Cholesterol Guidelines^10^, with specific attention to treatments targeting low-density lipoprotein cholesterol, to provide a framework for optimizing lipid lowering therapy in patients with known prior ASCVD.

### Basics of the lipid panel

A standard lipid panel provides total blood cholesterol levels as well as values for lipid subfractions. In general, the three main lipid subfractions of interest include low-density lipoprotein cholesterol (LDL-C), high-density lipoprotein cholesterol (HDL-C), and triglycerides (TG). On a standard lipid panel, total cholesterol, HDL-C, and TG are all directly measured. By contrast, LDL-C is usually calculated by the Friedwald formula (LDL-C = Total Cholesterol –TG/5 –HDL-C), unless the triglycerides are markedly elevated (> 4.5 mmol/L, 400 mg/dL) or LDL-C is very low (< 1 mmol/L, 40 mg/dL), in which case alternative formulas or methods are used ^11–13^. Fasting and non-fasting lipid samples are equally adequate for most clinical applications^14^. However, in patients who demonstrate hypertriglyceridemia, a fasting lipid panel is recommended to confirm the diagnosis^15,16^.

Abnormalities of the lipid panel such as high LDL-C, low HDL-C, and high TG are all associated with greater ASCVD risk^17–19^. Several large cardiovascular outcomes trials have assessed the clinical benefit of modifying each type of lipid subfraction but have been met with varying results. Randomized controlled trials have repeatedly demonstrated that reduction of LDL-C decreases the incidence of cardiovascular events in both primary and secondary prevention cohorts^20^. On the other hand, attempts at increasing HDL-C with pharmacologic therapy have failed to show consistent benefit towards a composite cardiovascular endpoint^21–27^. Similarly, evidence supporting the use of TG-modifying therapies such as fibrates, niacin, or omega-3 fatty acids for ASCVD risk reduction has been mixed, especially in patients on baseline LDL-lowering therapy. High dose icosapent ethyl, a highly purified form of the omega-3 fatty acid eicosapentaenoic acid, has been shown to reduce major adverse cardiac events in statin-treated patients with elevated TG and residual risk for ASCVD^28^. However, the benefits were independent of baseline or on-treatment TG levels suggesting the role of additional pleotropic effects of the drug^29^.

### Potency of LDL-C modifying therapies

Given the robust data favoring LDL-C reduction, guideline-based management of lipids in patients with established ASCVD is centered around LDL-C lowering therapies. The five standard LDL-C modifying medication classes available in clinical practice today include statins, ezetimibe, bile acid sequestrants, proprotein convertase subtilisin kexin type 9 (PCSK9) inhibitors, and bempedoic acid. These medication classes differ in their mechanism of action and their ability to achieve optimal LDL-C levels (Table 1).

**Table 1:** Percent of LDL-C-lowering provided by various lipid modifying therapies. Bempedoic acid is more likely to be used in statin-intolerant patients and provides greater LDL-C percent reduction in this setting. A combination pill of bempedoic acid and ezetimibe is currently available that further lowers LDL-C by 35% in patients on stable background statin therapy.

**Treatment Category**	**Examples**	**LDL-C % Reduction**
**High intensity statin**	Atorvastatin 40–80 mg daily	≥50%
	Rosuvastatin 20–40 mg daily	
**Moderate intensity statin**	Atorvastatin 10–20 mg daily	30–49%
	Fluvastatin 80 mg daily	
	Lovastatin 40 mg daily	
	Pitavastatin 2–4 mg daily	
	Pravastatin 40–80 mg daily	
	Rosuvastatin 5–10 mg daily	
	Simvastatin 20–40 mg daily	
**Low intensity statin**	Fluvastatin 20–40 mg daily	< 30%
	Lovastatin 20 mg daily	
	Pitavastatin 1 mg daily	
	Pravastatin 10–20 mg daily	
	Simvastatin 10 mg daily	
**Ezetimibe**	Ezetimibe 10 mg daily	20–25% when added to baseline statin therapy
**Bile Acid Sequestrants**	Colesevelam 3.75 g daily	15% when added to baseline statin therapy
	Cholestyramine 4–24 g daily	15–25% when not receiving statin therapy
**PCSK9 Inhibitor**	Evolocumab 140 mg q2 weeks	50–70% when added to baseline statin therapy
	Alirocumab 75 mg q2 weeks	
**Bempedoic Acid**	Bempedoic acid 180 mg daily	16–18% when added to baseline statin therapy
		20–22% when not receiving statin therapy
**Bempedoic Acid + Ezetimibe Combination**	Bempedoic acid 180 mg + Ezetimibe 10 mg daily	35% when added to baseline statin therapy
**Inclisiran**	Inclisiran 300 mg on day 1, day 90, then q6 months	50% when added to baseline statin therapy

Statins are competitive, reversible inhibitors of 3-hydroxy-3-methylglutaryl coenzyme A (HMG-CoA) reductase and continue to be the primary treatment modality for guideline-based management of lipids in patients with ASCVD^30^. Through inhibiting HMG-CoA reductase, statins halt the rate limiting step of hepatic endogenous cholesterol production. A decrease in liver cholesterol leads to upregulation of the LDL-receptors on hepatocytes, facilitating endocytosis of LDL-C and its precursors from the systemic circulation, thereby reducing LDL-C levels in the bloodstream^31^.

Statin regimens can be subdivided into low-intensity, moderate-intensity, and high-intensity subgroups based on the percent reduction of LDL-C achieved at that specific dose and formulation. Most of the LDL-C reducing effect is seen with the initial starting dose. Each additional doubling of the dose of any statin only provides an additional 6% reduction in LDL-C^32^. For example, though 10 mg of atorvastatin reduces LDL-C by 37%, sequential doubling of this dose to 20 mg, 40 mg, and 80 mg provides 43%, 49%, and 55% LDL-C reduction, respectively. There are at least two likely explanations for the decreased efficacy with higher doses. First, as endogenous cholesterol biosynthesis is inhibited, there is a compensatory increase in exogenous cholesterol absorption through the gastrointestinal tract^33^. Second, statin treatment induces production of PCSK9, a key protein that binds hepatic LDL receptors, triggers receptor degradation, and prevents clearance of LDL-C particles from the circulation^34^.

Ezetimibe, an oral selective cholesterol absorption inhibitor, is the most commonly used non-statin agent. Combined treatment with statin and ezetimibe synergistically targets both the endogenous and exogenous pathways of cholesterol metabolism. When added to baseline statin therapy, ezetimibe provides an additional 20–25% reduction in LDL-C^33,35^. Whereas 80 mg atorvastatin provides 55% LDL-C reduction, a similar reduction can be achieved by 10 mg atorvastatin plus 10 mg ezetimibe.

Bile acid sequestrants, oral polymers that impede the reabsorption of bile salts in the small intestine, represent another non-statin alternative that have proven LDL-C lowering efficacy. Depending on the dose prescribed, bile acid sequestrants reduce LDL-C levels by 15–25%^36–38^, and are felt to have a favorable safety profile due to a lack of meaningful absorption from the gut into the bloodstream. However, gastrointestinal complaints, high pill burden, drug-drug interactions, and lack of outcomes data in the statin era have limited the use of this class of medications in modern clinical practice.

PCSK9 inhibitors prevent the PCSK9 protein from binding to the LDL receptor, thereby interrupting attempts at LDL receptor degradation and allowing for increased clearance of LDL-C from the bloodstream. The currently available PCSK9 inhibitors are injectable monoclonal antibodies that markedly reduce LDL-C and are increasingly used in high-risk patients. In statin-treated patients, PCSK9 inhibitors have been shown to reduce LDL-C levels by an additional 50–70%^39,40^.

Bempedoic acid was granted approval by the US Food and Drug Administration (FDA) in February 2020 as an adjunct to maximally tolerated statin therapy in adults with familial hypercholesterolemia or established ASCVD who require additional lowering of LDL-C^41^. Bempedoic acid is an oral prodrug that undergoes liver-specific activation to inhibit adenosine triphosphate-citrate lyase, an important enzyme in the cholesterol biosynthesis pathway directly upstream of HMG-CoA reductase. In multiple phase 3 trials, treatment with bempedoic acid has been shown to lower LDL-C levels by 16–18% in patients on maximally tolerated statin therapy^42–45^. A combination of bempedoic acid and ezetimibe reduces LDL-C by approximately 35%^46^, and is a promising alternative in patients who are intolerant of statins.

### Evidence base for LDL-modifying therapies

Clear evidence supporting reduction of LDL-C with statins to lower the incidence of major cardiovascular events comes from a 2010 meta-analysis performed by the Cholesterol Treatment Trialists’ (CTT) Collaboration. Given the understanding that small sample sizes, inadequate event rates, and short follow up are all likely to lead to underpowered and negative individual studies, the CTT Collaboration was founded to meta-analyze all randomized controlled trials of statin therapy that studied at least 1,000 patients over 2 years of treatment with the aim of quantifying the effect of LDL-C lowering on major cardiovascular events.

**Figure 1.**
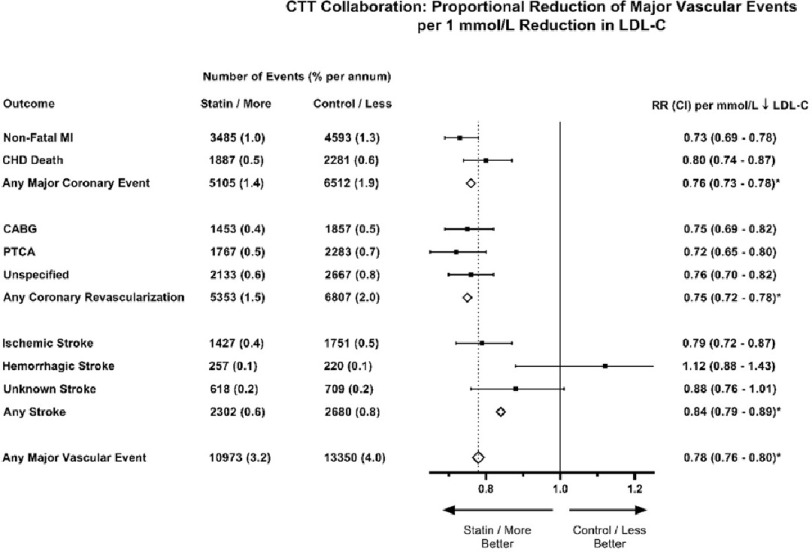
Summary of major vascular event endpoints in CTT Collaboration per one mmol/L reduction in LDL-C. Adapted from the CTT Collaboration website (https://www.cttcollaboration.org/). Each one mmol/L reduction in LDL-C was associated with significant relative risk reductions in major coronary events (24%), coronary revascularizations (25%), and stroke (16%). Altogether, there was a 22% relative risk reduction across all major vascular events per one mmol/L reduction in LDL-C. All confidence intervals are 95% confidence intervals unless indicated by an asterisk, in which a 99% confidence interval is shown. MI = Myocardial Infarction. CHD = Coronary Heart Disease. CABG = Coronary Artery Bypass Grafting. PTCA = Percutaneous Transluminal Coronary Angioplasty. RR = Relative Risk. CI = Confidence Interval. LDL-C = Low-Density Lipoprotein Cholesterol.

In an analysis of 26 randomized trials involving nearly 170,000 individuals, major vascular event rates were directly related to the absolute reduction in LDL-C that was achieved^20^. Specifically, each one mmol/L (∼38.7 mg/dL) reduction in LDL-C with statin therapy produced a 22% proportional reduction in the rate of major vascular events over a median follow up of five years (Figure 1). This finding was remarkably consistent across baseline LDL-C and various subgroups, including those with and without prior ASCVD. Moreover, across all trials, the absolute reduction in LDL-C levels while on treatment accounted for 98% of the between-study variability in relative risk reduction^47^.

This same relationship between achieved reduction in LDL-C and relative risk reduction for ASCVD events has been shown to be equally true for non-statin therapies. In a meta-analysis of eight trials studying diet, ezetimibe, bile acid sequestrants, and ileal bypass, each one mmol/L reduction in LDL-C was associated with a 25% relative risk reduction in cardiovascular event rates^47^. Though similar analyses of PCSK9 inhibitor trials are limited by short duration of follow up, PCSK9 inhibitor treatment provides consistent effects on the risk of cardiovascular events per mmol/L reduction in LDL-C when directly comparing the first years of treatment^48,49^.

Implicit in this finding is the fact that if all individuals receive an equivalent relative risk reduction per mmol/L reduction in LDL-C, then those with higher absolute risk are most likely to receive the greater absolute benefit. For example, a low risk, young individual without cardiovascular risk factors with a 10-year ASCVD risk of 1% would reduce their risk to approximately 0.78% risk if they achieved one mmol/L LDL-C lowering over the time period studied. A higher risk, older individual with cardiovascular comorbidities at 30% baseline 10-year risk experiencing an equivalent LDL-C reduction would have 23.4% risk over the same period and receive a greater absolute benefit (0.22% vs. 6.6% per decade).

Additional data suggest that the relative risk reduction provided by LDL-C-lowering is even greater when initiated earlier in life. Mendelian randomization studies of individuals who inherit an LDL-C lowering allele show that long-term exposure to lower LDL-C through a genetic mutation is associated with a three-fold greater reduction in the risk of ASCVD per one mmol/L decrease in LDL-C when compared to the same degree of LDL-C-lowering in adulthood^50^. These data suggest that LDL-C has not only a causal but possibly a cumulative effect on the risk of ASCVD. Taken together, reducing LDL-C levels leads to a dose-dependent decrease in the risk of major ASCVD events that is directly proportional to the absolute magnitude of reduction achieved in LDL-C, with a possible larger effect seen over larger periods of time^51^.

### Guideline-based management of lipids in secondary prevention of atherosclerotic cardiovascular disease

The 2018 multi-society Blood Cholesterol Guidelines define clinical ASCVD as an all-encompassing term for several diseases of atherosclerotic origin^10^. Patients with acute coronary syndromes, prior MI, stable angina, stroke, transient ischemic attack, peripheral artery disease, or history of any arterial revascularization due to atherosclerotic disease are at very high risk for future ischemic events. All patients with clinical ASCVD are recommended to be on lipid lowering therapy.

### All patients with ASCVD should be considered for maximally tolerated statin therapy

In patients with clinical ASCVD ≤75 years of age, high-intensity statin therapy is recommended with the goal of achieving a 50% or greater reduction in LDL-C levels. If high-intensity statin therapy is contraindicated or limited by side effects, a moderate-intensity statin is supported.

In the CTT Collaboration meta-analysis, there were 21 trials that compared statin versus control, and another five trials that compared more versus less intensive statin regimens^20^. Among the 21 trials that assessed allocation to routine statin therapy versus no statin therapy, the average reduction in LDL-C was approximately one mmol/L, which resulted in a 22% relative risk reduction in major cardiovascular events. In five trials comparing more (i.e., high-intensity) versus less intensive (i.e., moderate-intensity) statin therapy, the mean additional reduction in LDL-C was 0.51 mmol/L (19.7 mg/dL), which translated into a further 15% relative risk reduction in major vascular events.

**Figure 2.**
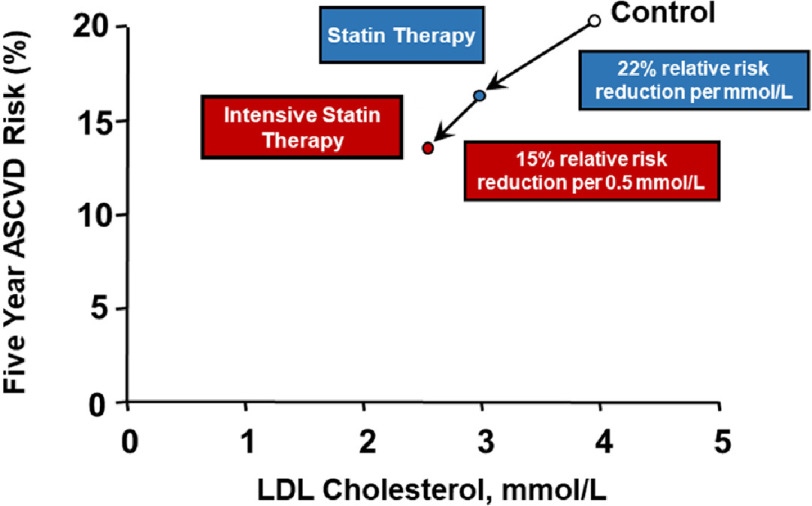
High intensity statin therapy provides additional relative risk reduction to moderate intensity statin therapy. Adapted from the CTT Collaboration website (https://www.cttcollaboration.org/). On average, allocation to moderate-intensity statin therapy reduces LDL-C by approximately one mmol/L and leads to a 22% relative risk reduction in major vascular events. Treatment with an intensive (i.e., high-intensity) statin regimen leads to an additional 0.5 mmol/L reduction in LDL-C and further lowers the risk of major vascular events by an additional 15%. It follows that a patient with 20% baseline five year ASCVD risk would reduce risk to 16% (20 ×0.78) with moderate-intensity statin therapy, or 13% (20 ×0.78 ×0.85) with high-intensity statin therapy over the time period studied. In patients with prior ASCVD, guidelines support maximal risk reduction with the most intensive statin treatment tolerated.

Combining the evidence in the intention-to-treat analyses of these two groups of trials revealed that allocation to a high-intensity statin regimen reduces LDL-C by approximately 1.5 mmol/L (58.4 mg/dL) when compared to no statin therapy^52^. Therefore, it is estimated that treatment with a high-intensity statin provides a 34% relative risk reduction for major vascular events over the time period studied (0.78 × 0.85 = 0.66). In terms of absolute risk, an individual with prior ASCVD at baseline 20% five-year risk of a major vascular event would have a reduction in the predicted 5-year event rate to 13% (20 × 0.66) if treated with a high-intensity statin, or to 16% (20 × 0.78) if treated with a moderate-intensity statin (Figure 2). Based on these data, guidelines support the initiation of the highest intensity statin tolerated.

In patients with age greater than 75 years, the 2018 multi-society Blood Cholesterol Guidelines recommend initiation of a moderate or high intensity statin after weighing these benefits against adverse effects^10^. In contrast, the 2019 European Society of Cardiology (ESC)/European Atherosclerosis Society (EAS) Guidelines recommend treatment with the highest intensity statin tolerated across all age ranges^53^. The primary reason behind this discrepancy had been the observation in the CTT Collaboration data that the relative reduction in major vascular events per one mmol/L LDL-C reduction was possibly lower among patients aged > 75 years^54^. However, in a meta-analysis of all recent trials of LDL-C-lowering therapy, those with age ≥75 years experienced a 26% relative risk reduction per one mmol/L lowering of LDL-C, with no significant difference from those younger than 75 years^55^. Given this clearer data that LDL-C lowering is as effective in reducing major cardiovascular events in patients aged 75 years or older, and the knowledge that the absolute risk of ASCVD rises with increasing age, it is expected that future guidelines will strengthen recommendations for lipid lowering in this older population.

### Ezetimibe should be considered in patients with ASCVD and LDL-C Level of 1.8 mmol/L (70 mg/dL) or higher

In patients with prior ASCVD who continue to have elevated LDL-C levels on statin monotherapy, adjunctive therapy with ezetimibe is recommended. The addition of ezetimibe to baseline statin therapy provides additional reduction in LDL-C and is a Class II recommendation in the Blood Cholesterol Guidelines. Ezetimibe use in this population is supported by the results of The Improved Reduction of Outcomes: Vytorin Efficacy International Trial (IMPROVE-IT). In IMPROVE-IT, 18,144 patients with ACS in the prior 10 days who had LDL-C levels of 1.3–2.6 mmol/L (50–100 mg/dL) on lipid lowering therapy or 1.3–3.2 mmol/L (50-125 mg/dL) without lipid lowering therapy were randomized to receive either combination simvastatin 40 mg-ezetimibe 10 mg versus simvastatin 40 mg alone^56^. The primary endpoint was a composite of cardiovascular death, nonfatal MI, nonfatal stroke, unstable angina requiring hospitalization, and coronary revascularization greater than 30 days after randomization.

At time of hospitalization for the index event, the mean LDL-C was 2.4 mmol/L (93.8 mg/dL) in both groups. Patients in the combined simvastatin-ezetimibe arm had a median time-weight average LDL-C of 1.4 mmol/L (53.7 mg/dL) whereas those in the simvastatin alone arm had a median time-weighted average LDL-C of 1.8 mmol/L (69.5 mg/dL). At seven years, the primary endpoint occurred less frequently in the combined treatment arm versus the statin monotherapy arm (32.7% vs. 34.7%, HR 0.936, 95% CI [0.89–0.99], *p* = 0.016, number needed to treat = 50). The relative risk reduction in major cardiovascular events provided by ezetimibe was consistent with the prior CTT Collaboration finding of 22% reduction in major vascular events per one mmol/L reduction in LDL-C, as the HR for clinical benefit per one mmol/L LDL-C reduction in the study was 0.80. Of note, whereas the average baseline LDL-C level in the CTT Collaboration meta-analysis was 3.4 mmol/L (131 mg/dL), IMPROVE-IT demonstrated that the magnitude of clinical benefit from further LDL-C lowering was similar even at lower starting ranges of LDL-C. Furthermore, cardiovascular event rates were lowest in those who achieved the lowest LDL-C levels, with no observed excess in safety events even among those who achieved an LDL-C < 0.8 mmol/L (30 mg/dL) and were followed for 6 years^57^.

In IMPROVE-IT, there was a broad spectrum of risk in the trial with regard to the incidence of recurrent ischemic events. Patients with a greater burden of atherothrombotic risk factors not only had higher event rates, but also experienced greater benefit from ezetimibe, whereas those with fewer risk factors had lesser reductions in major cardiovascular outcomes^58^. High risk subgroups independently shown to have significantly larger benefits with ezetimibe than the rest of the study population included age ≥75 years^59^, prior stroke^60^, prior coronary artery bypass grafting^61^, diabetes^62^, and peripheral arterial disease^63^. As such, the most recent Blood Cholesterol Guidelines make a Class IIa recommendation for initiating ezetimibe in those with prior ASCVD, LDL-C greater than 1.8 mmol/L (70 mg/dL) on maximally tolerated statin therapy, and very high atherothrombotic risk due to risk-enhancing features. As the benefit of ezetimibe was less apparent in low risk trial participants, ezetimibe therapy in all individuals with prior ASCVD has received a weaker Class IIb recommendation.

### PCSK9 inhibitors should be considered in high-risk patients with ASCVD on maximal LDL-lowering therapy and LDL level of 1.8 mmol/L (70 mg/dL) or higher

In patients with prior ASCVD who are unable to achieve LDL-C levels of 1.8 mmol/L (70 mg/dL) with statins and ezetimibe, treatment with PCSK9 inhibitors is guideline supported for the highest risk patients. The data for intensive lipid lowering with PCSK9 inhibitors comes from two large cardiovascular outcomes trials. The two agents studied in these trials, evolocumab and alirocumab, are fully human monoclonal antibodies administered subcutaneously and have been approved for clinical use^49^.

The Further Cardiovascular Outcomes Research with PCSK9 Inhibition in Subjects with Elevated Risk (FOURIER) trial was the first successfully completed cardiovascular outcomes trial of PCSK9 inhibition. In FOURIER, 27,564 high-risk patients with established cardiovascular disease, defined as prior MI, prior non-hemorrhagic stroke, or symptomatic PAD, were recruited into a lipid stabilization phase which included intensive statin therapy with or without ezetimibe^64^. Those on maximal medical therapy who continued to have LDL-C ≥1.8 mmol/L (70 mg/dL) or non-HDL-C ≥2.6 mmol/L (100 mg/dL) were then randomly assigned to receive evolocumab or matching placebo injections. The primary endpoint was a composite of major cardiovascular events. The median duration of follow up was 2.2 years.

**Figure 3.**
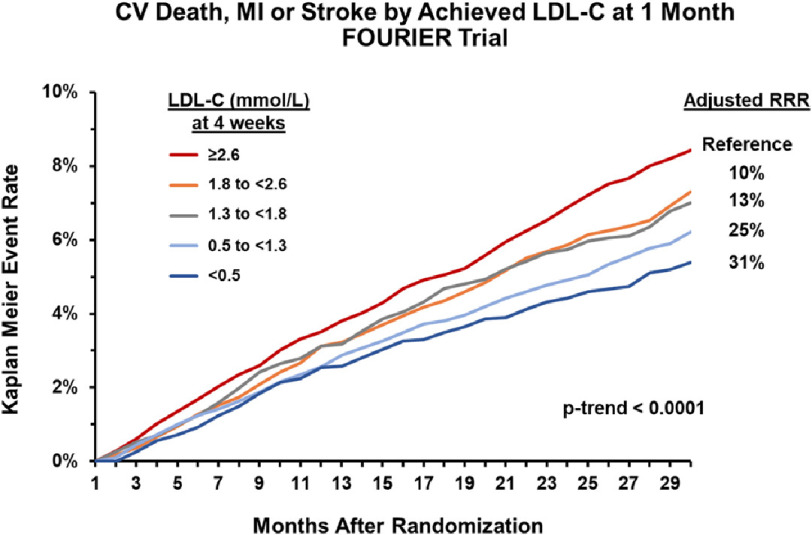
Kaplan-Meier event rate of cardiovascular death, myocardial infarction or stroke in FOURIER trial by achieved LDL-C at 4 weeks. In the FOURIER trial^[64]^, the achieved LDL-C at four weeks following randomization was significantly associated with incidence of the key secondary endpoint, a composite of cardiovascular death, myocardial infarction, or stroke. Groups that achieved lower LDL-C targets obtained greater relative risk reduction (p-trend< 0.0001). The group of subjects that achieved LDL-C < 0.5 mmol/L (20 mg/dL) had the lowest incidence of the combined endpoint, and had a 31% relative risk reduction compared to the reference group (LDL ≥2.6 mmol/L, 100 mg/dL) over the trial study period. CV = Cardiovascular. MI = Myocardial Infarction. LDL-C = Low-Density Lipoprotein Cholesterol. RRR = Relative Risk Reduction.

The median baseline plasma LDL-C level for the study was 2.4 mmol/L (92 mg/dL). Evolocumab lowered LDL-C levels by 59% at 48 weeks compared to placebo, with a median on-treatment level of 0.8 mmol/L [IQR 0.5 to 1.2, 30 mg/dL (IQR: 19 to 46)], a reduction that was maintained over the duration of the study. The evolocumab treatment group had a significantly lower incidence of the primary composite endpoint of cardiovascular death, nonfatal MI, nonfatal stroke, hospitalization for unstable angina, or coronary revascularization when compared to placebo (9.8% vs 11.3%; HR 0.85, 95% CI [0.79–0.92], *p* < 0.001). The benefit of evolocumab was consistent across all key subgroups, including the patients in the lowest quartile of baseline LDL-C levels (< 1.9 mmol/L, 74 mg/dL) and those on maximal dose statin^65^. Moreover, on-treatment LDL-C at four weeks was highly predictive of risk for the primary and secondary composite endpoints, with incremental risk reduction conferred to subgroups that achieved lower targets (p-trend < 0.0001, Figure 3)^66^.

ODYSSEY OUTCOMES was a second randomized controlled trial of PCSK9 inhibition that studied 18,924 patients with recent acute coronary syndromes in the prior 1 to 12 months^67^. Patients first underwent a run-in period involving stabilization on the highest tolerated dose of atorvastatin or rosuvastatin. Those with LDL-C ≥1.8 mmol/L (70 mg/dL), non-HDL-C ≥2.6 mmol/L (100 mg/dL), or apolipoprotein B ≥2.1 mmol/L (80 mg/dL) while on maximally tolerated statin treatment qualified for randomization to either alirocumab or placebo subcutaneous injections every two weeks. Subjects assigned to the alirocumab treatment arm underwent a blinded dose-adjustment protocol to target an LDL-C range of 0.6–1.3 mmol/L (25-50 mg/dL) during this study. Though on-treatment LDL-C levels ≤0.6 mmol/L (25 mg/dL) were permitted, patients who achieved sustained LDL-C levels below 0.4 mmol/L (15 mg/dL) while on the lowest dose of alirocumab were switched to placebo. Patients were followed for a median of 2.8 years for the primary composite endpoint of coronary-related death, nonfatal MI, nonfatal ischemic stroke, or unstable angina requiring hospitalization.

The mean LDL-C across both treatment groups at baseline was 2.4 mmol/L (92 mg/dL). When compared to placebo, alirocumab treatment reduced LDL-C levels by 63%, 61%, and 55% at 4, 12, and 48 months, respectively, among those who remained on treatment. In an intention-to-treat analysis, the composite primary endpoint occurred less frequently in the alirocumab treatment arm compared to placebo (9.5% vs 11.1%, HR 0.85, 95% CI [0.78–0.93], *p* < 0.001). Subgroup analyses demonstrated that those who had a baseline LDL-C ≥2.6 mmol/L (100 mg/dL) may have had a more prominent absolute risk reduction than those with lower baseline LDL-C levels. However, due to the protocol mandated dose-adjustment algorithm, in which patients with lower baseline LDL-C levels were more likely to receive the lower dose of alirocumab and more likely to have alirocumab stopped completely due to very low achieved LDL-C, the observation that subgroups with lower LDL-C levels at baseline received a diminishing degree of LDL-C reduction is likely to be confounded.

From the results of these two cardiovascular outcomes trials, the 2018 Blood Cholesterol Guidelines recommend consideration of PCSK9 inhibitors in high-risk patients with prior ASCVD events on maximally tolerated LDL-C lowering therapy and LDL ≥1.8 mmol/L (70 mg/dL). The decision to initiate treatment should weigh the expected benefit towards major adverse cardiovascular events against the expenses incurred from being on treatment (approximately $6,000/year in the USA), though expected future reductions in the price of these drugs will allow this therapy to be more cost-effective with time^68^. Additionally, though five year follow up from an open-label extension of phase 2 studies with evolocumab raised no new safety concerns, longer-term safety data from randomized trials are still needed^69^. Fortunately, two ongoing trials of PCSK9 inhibition with planed four to five-year follow up are underway (discussed below).

### Bempedoic acid may emerge as an alternative in statin-intolerant patients

Given the incorporation of non-statin LDL-C-lowering therapies into the professional guidelines, recent attention has been directed to bempedoic acid, a first-in-class orally administered ATP citrate lyase inhibitor which offers promise for additional LDL-C reduction in patients on maximally tolerated lipid-lowering therapy. Since the publication of the 2018 Blood Cholesterol Guidelines, several phase 3 lipid-lowering trials have shown that bempedoic acid significantly lowers LDL-C levels when compared to placebo in at-risk populations for which additional LDL-C lowering is recommended. While cardiovascular outcomes data for this drug class are not yet available, two large trials of bempedoic acid designed to study intermediate endpoints, CLEAR Harmony^42^ and CLEAR Wisdom^43^, had promising results reported in 2019.

CLEAR Harmony was a multicenter, randomized, placebo-controlled trial of 2,230 patients with either prior ASCVD, heterozygous familial hypercholesterolemia, or both and an LDL-C concentration of at least 1.8 mmol/L (70 mg/dL) while on maximally-tolerated lipid lowering therapy^42^. At enrollment, nearly all patients were on a statin and 7.7% were receiving treatment with ezetimibe. The mean LDL-C at baseline across the entire trial cohort was 2.7 mmol/L (103.2 mg/dL). Patients were randomized in a 2:1 fashion to treatment with 180 mg bempedoic acid daily or matching placebo for a total of 52 weeks. The primary endpoint was safety and the principal efficacy endpoint was the percentage change in LDL-C levels at 12 weeks. At week 12, treatment with bempedoic acid reduced LDL-C by 18.1% when compared to the placebo group (95% CI −20.0% to −16.1%, *p* < 0.001). This effect did not attenuate over the 52-week trial period.

CLEAR Wisdom was a similarly designed multicenter, randomized, placebo-controlled trial of 779 patients with prior ASCVD, heterozygous familial hypercholesterolemia, or both with an LDL-C concentration of at least 2.6 mmol/L (100 mg/dL) while on maximally-tolerated lipid lowering therapy at the first screening visit^43^. Unlike CLEAR Harmony, which was designed for a primary safety endpoint, CLEAR Wisdom was designed to assess the primary end point of percent change from baseline in LDL-C level at week 12. Most patients enrolled (89.6%) were on background statin therapy, but 5% of participants were not taking any lipid-lowering therapy at baseline. The mean LDL-C concentration at randomization was 3.1 mmol/L (120.4 mg/dL). Patients were randomized 2:1 to treatment with bempedoic acid or matching placebo. At week 12, treatment with bempedoic acid resulted in a 17.4% decrease in LDL-C concentration compared to placebo (95% CI −21.0% to −13.9%, *p* < 0.001). Additionally, bempedoic acid reduced levels of high-sensitivity C-reactive protein (hsCRP) throughout the study, consistent with other preclinical studies of the drug^70,71^.

Based on these data, bempedoic acid was granted FDA approval in February 2020 as an adjunctive treatment for patients with familial hypercholesterolemia or established ASCVD who require additional lowering of LDL-C. It is important to note that neither CLEAR Harmony nor CLEAR Wisdom was powered to study the effect of bempedoic acid on cardiovascular outcomes, so the expected clinical benefit of bempedoic acid is currently based on extensive prior data demonstrating a consistent linear relationship between achieved LDL-C levels and subsequent cardiovascular risk. While it is notable that adjudicated major adverse cardiovascular event rates were lower in the bempedoic acid treatment arms in both trials, these were trends that did not reach statistical significance. CLEAR Outcomes, a large phase 3 clinical outcomes trial with bempedoic acid, is ongoing with results anticipated in 2022-23 (discussed below)^72^.

### Lipoprotein apheresis can be considered for additional LDL-C lowering in patients who have inadequate response to available therapies

In high-risk patients in whom the above therapeutic options are not tolerated, or ineffective in achieving target LDL-C levels, lipoprotein apheresis remains a highly-effective rescue treatment for severe dyslipidemia^73^. In lipoprotein apheresis, the extracorporeal removal of circulating apoB-containing lipoproteins acutely reduces LDL-C levels, lowering LDL-C by 60–80% when compared to concentrations immediately prior to the procedure. However, given the transient nature of these LDL-C reductions, serial treatments every one or two weeks are necessary to achieve the degree of time-averaged lowering of LDL-C desired^74,75^.

Apheresis represents a highly-specialized, time-intensive, and expensive endeavor to achieve LDL-C targets in patients, with costs estimated at $50,000 per year^76^. For these reasons, extracorporeal therapy is usually reserved for patients with homozygous familial hypercholesterolemia in whom lipid-lowering drugs are usually less effective, and LDL-C reduction can be life-saving^77,78^. Lipoprotein apheresis is also offered to patients with heterozygous or severe forms of hypercholesterolemia, in whom maximal lipid-lowering therapy only reduces LDL-C to levels of 5.2–7.8 mmol/L (200-300 mg/dL)^79,80^. The LDL-C level at which to initiate treatment varies by country and is usually influenced by whether ASCVD is present. As such, the need for lipoprotein apheresis is often determined by specialists on a case-by-case basis.

The reason behind such varied practice internationally is due to the lack of randomized longitudinal outcomes data for lipoprotein apheresis. Observational studies comparing lipoprotein apheresis to standard oral drug therapy have suggested that extracorporeal treatment reduces a composite cardiovascular endpoint by 72% over a six year follow up period^81^. Retrospective analyses comparing the burden of ASCVD before and after initiation of apheresis therapy have suggested a reduction in cardiovascular events between 30–80% in the time period on apheresis treatment^82,83,84^.

Though lipoprotein apheresis has had a larger role in prior decades, recently approved treatments such as PCSK9 inhibitors may decrease the necessity of apheresis in contemporary practice. Data supporting this claim comes from the ODYSSEY ESCAPE trial, a double-blind, randomized, placebo-controlled trial of PCSK9 inhibition in patients with heterozygous familial hypercholesterolemia who were undergoing weekly or biweekly lipoprotein apheresis^85^. In this trial, a total of 62 subjects were randomly assigned in a 2:1 fashion to alirocumab 150 mg every 2 weeks or matching placebo for an 18 week follow up period. In weeks 1 through 6, the apheresis rate was fixed while patients initiated the randomly assigned treatment. In weeks 7 through 18, apheresis treatments were adjusted based on the LDL-C response in a blinded manner, with complete discontinuation of apheresis if the LDL-C was reduced by 30% or more from baseline. The primary efficacy endpoint was the change in rate of apheresis treatments over a 12-week (weeks 7 through 18) follow up period.

At the end of week 6, the change in LDL-C from baseline was -53.7% (95% CI −58.2 to −49.2) in the alirocumab group and 1.6% (95% CI −4.7 to 7.9) in the placebo group. From weeks 7 through 18, patients assigned to alirocumab experienced a 75% reduction in the standardized rates of apheresis when compared to placebo-treated patients (*p* < 0.0001). More specifically, 90% of patients on alirocumab halved the rate of apheresis over the 12-week follow up period, while 63.4% of patients on alirocumab avoided apheresis altogether. Based on the results of this study, PCSK9 inhibitors may play an increasing role in patients with heterozygous familial hypercholesteremia undergoing routine apheresis therapy. Furthermore, with the recent approval of bempedoic acid, and many other investigational LDL-C lowering therapeutics in development, it is possible that the need for apheresis may be decreased further in the coming years.

### Safety of LDL-C lowering therapies

Although the benefits of LDL-C lowering therapies are well documented, no drug class is without adverse effects and all decisions regarding initiation of treatment should weigh the risks and benefits for individual patients.

Statins cause a small absolute excess of muscle related adverse events (approximately 1 case per 10,000 individuals treated per year)^52,86,87^, with increased rates at higher doses of statins and when used concurrently with drugs that affect their metabolism^88^. In observational studies, statin treatment has been associated with a higher rate of symptomatic muscle pain or weakness in roughly 5% of patients^89,90^, but these rates have been shown to be equivalent in placebo-treated patients across numerous randomized controlled trials^91,92^. It is also estimated that standard statin regimens increase the risk of incident diabetes by approximately 10%^93^, though the clinical relevance of this finding is unclear given the proven cardiovascular benefits of statin therapy in patients with normal glucose homeostasis, glucose intolerance, and diabetes. In the CTT meta-analysis, every one mmol/L reduction in LDL-C by statin therapy resulted in a non-significant increase in hemorrhagic stroke (RR 1.12, 95% CI [0.93–1.35], *p* = 0.2), but this was offset by a greater reduction in ischemic strokes for the same per unit change in LDL-C (RR 0.79, 95% CI [0.74–0.85], *p* < 0.0001)(Figure 1)^20^. Observational reports have sporadically linked statins with cognitive decline, cataracts, and cancer, but these observations have been refuted in multiple large randomized controlled studies^20,94–97^.

Ezetimibe is generally well tolerated, and in IMPROVE-IT there were no differences between the study groups in any of the prespecified safety endpoints or rates of drug discontinuation^56^. Isolated reports of ezetimibe-associated upper respiratory tract infection, diarrhea, myalgias and arthralgias exist, but are rare and have not been seen in significant excess in any clinical studies^98^.

Bempedoic acid is a prodrug that is exclusively metabolized in the liver and therefore may avoid the muscle-specific adverse effects associated with statin therapies. In CLEAR Harmony, the incidence of adverse events did not differ between the bempedoic acid and placebo groups. However, the bempedoic acid group did have a higher rate of adverse events leading to discontinuation of the assigned drug (10.9% vs 7.1%, *p* = 0.005) and a higher incidence of gout (1.2% vs. 0.3%, *p* = 0.03)^42^, likely explained by bempedoic acid byproducts competing for uric acid transporters in the kidney. Combination of the CLEAR Harmony and CLEAR Wisdom trials revealed that the risk of gout events was even higher in those with a prior history of gout (11.2% bempedoic acid vs 1.7% placebo), providing caution for use in this specific subgroup of patients^42,43^. Bempedoic acid may affect other lipid parameters, and a meta-analysis of 10 randomized trials of bempedoic acid therapy has noted a 6% decrease in HDL-C among those on treatment^99^. Additionally, the FDA labeling for bempedoic acid warns of possible increased risk of tendon rupture with the drug, as early clinical trial experience demonstrated tendon rupture occurred in 0.5% of 2,009 patients treated with bempedoic acid versus 0% of 999 patients treated with placebo.

PCSK9 inhibitors appear to have a promising safety profile. For the two PCSK9 inhibitors approved for clinical use by the FDA (evolocumab, alirocumab), treatment has not been shown to increase the rates of adverse events, serious adverse events, or adverse events leading to drug discontinuation. In both trials there was a higher rate of injection-site reactions, but the majority of these were mild and did not lead to drug discontinuation^64,67^. Prospective evaluation of cognition has revealed no effects on executive function, memory, or psychomotor speed between the PCSK9 inhibitor evolocumab and placebo^100,102^. A similar study with alirocumab is ongoing (NCT02957682), though no difference in neurocognitive event rates from ODYSSEY OUTCOMES has been noted to date.

More broadly, PCSK9 inhibitors lead to profound LDL-C lowering and provide insight on the safety of lowering LDL-C beyond targets endorsed by current guidelines^65,101^. An analysis detailing the rates of safety events by achieved LDL-C at four weeks in the FOURIER trial showed no excess of multiple prespecified safety outcomes including increases of hepatic transaminases, creatinine kinase, neurocognitive events, new onset diabetes, cataracts, malignancy, hemorrhagic stroke, or non-cardiovascular death in patients achieving an LDL-C < 0.5 mmol/L (20 mg/dL) at 4 weeks compared to those with higher achieved LDL-C levels. Additionally, patients with ultra-low LDL-C concentrations less than 0.26 mmol/L (10 mg/dL) had similar rates of adverse events when compared to patients in the reference group of LDL-C ≥2.6 mmol/L (100 mg/dL)^66^.

### LDL-C treatment targets in secondary prevention have lowered over time

**Figure 4.**
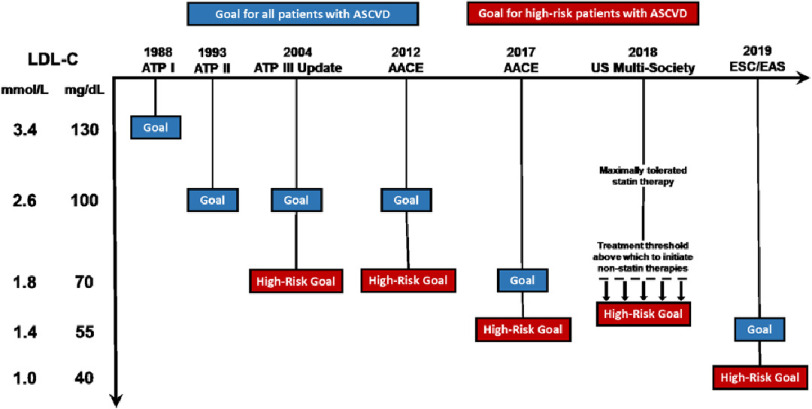
LDL-C treatment targets in cholesterol guidelines over time. LDL-C treatment targets were first suggested in 1988 by guidelines released by the Adult Treatment Panel and were broadly recommended for any individual at risk for ASCVD. In the three decades since, guidelines have suggested lower targets for those with established ASCVD and those who are at high risk for recurrent events. The most recent set of European guidelines from 2019 have recommended an LDL-C goal < 1.4 mmol/L (55 mg/dL) in high-risk populations, and a more aggressive target of < 1 mmol/L (40 mg/dL) in patients experiencing recurrent events. ATP = Adult Treatment Panel. AACE = American Association of Clinical Endocrinologists. ESC = European Society of Cardiology. EAS = European Atherosclerosis Society.

Given the mounting evidence that pharmacologic LDL-C lowering provides relative risk reduction in major adverse cardiovascular events directly proportional to the magnitude of LDL-C lowering, and the increasing data showing safety at very low LDL-C ranges, it should not be surprising to find that LDL-C treatment targets continue to decrease over time (Figure 4).

In 1985, the National Heart, Lung, and Blood Institute (NHLBI) established the Adult Treatment Panel (ATP), a panel of experts under the National Cholesterol Education Program seeking to detect, evaluate, and treat high blood cholesterol and reduce ASCVD risk in adults. The first set of cholesterol guidelines, named ATP-I, was published in 1988 and outlined a strategy for primary prevention for coronary heart disease in individuals with elevated levels of LDL-C^103^. The treatment strategy stated that a goal LDL-C of 3.4 mmol/L (130 mg/dL) in all individuals at risk for ASCVD was desirable. Notably, this first set of guidelines did not comment on secondary prevention in those with prior ASCVD events.

In the decades since, subsequent guidelines have incorporated targets for those with established ASCVD (ATP-II, 1993)^104^ and those deemed to be at very high risk for recurrent events (ATP-III, 2004)^105^. In the last five years, the American Association of Clinical Endocrinologists (AACE) and 2018 multi-society Blood Cholesterol Guidelines have supported the initiation of non-statin therapies to achieve LDL-C < 1.4 mmol/L (55 mg/dL) for those at high risk^10,106^. Most recently, the ESC/EAS has recommended an LDL-C goal < 1.4 mmol/L (55 mg/dL) for both primary and secondary prevention in high risk patients, with consideration of an even lower target of < 1 mmol/L (40 mg/dL) for patients experiencing repeated events^53^.

### Key ongoing cardiovascular outcome trials

**Figure 5.**
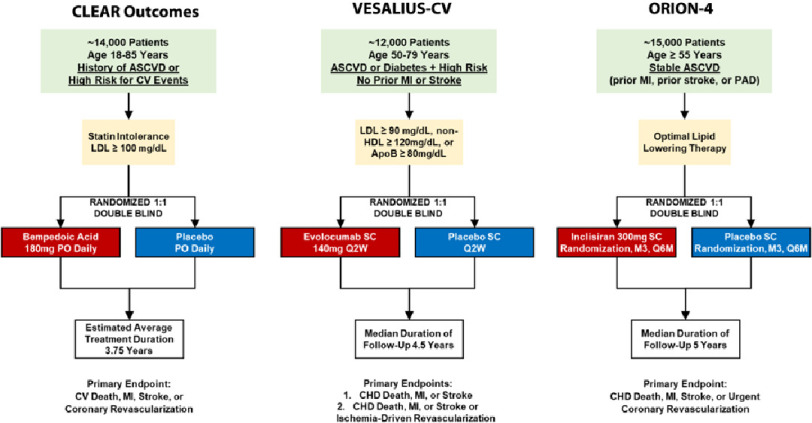
Key ongoing cardiovascular outcome trials of LDL-C lowering therapy. Three key trials are planned that are expected to inform clinical practice and impact future guidelines. CLEAR Outcomes (NCT02993406) will study the efficacy of bempedoic acid in reducing major cardiovascular events. VESALIUS-CV (NCT03872401) will assess the potential of PCSK9 inhibition in the prevention of a first-ever cardiovascular event. ORION-4 (NCT03705234) is investigating the clinical performance of inclisiran, an investigational cholesterol-lowering treatment.

Three important clinical outcomes trials are well underway that will add substantially to practice and may impact future guidelines (Figure 5).

CLEAR Outcomes (NCT02993406), the first phase 3 clinical outcomes trial studying bempedoic acid, has randomized 14,014 patients with statin intolerance, LDL-C ≥2.6 mmol/L (100 mg/dL), and high risk for ASCVD to either bempedoic acid 180 mg daily or matching placebo^72^. The primary endpoint is a composite of cardiovascular death, non-fatal MI, non-fatal stroke, or coronary revascularization and the estimated average treatment duration is 3.75 years. Results are anticipated in 2022-23.

VESALIUS-CV (NCT03872401), the next large trial of the PCSK9 inhibitor evolocumab, is designed to assess the efficacy of this therapy on the prevention of a first-ever cardiovascular event. Approximately 12,000 patients at high cardiovascular risk but no prior history of MI or stroke will undergo 1:1 randomization to subcutaneous evolocumab or matching placebo. Two separate primary composite cardiovascular outcomes will be measured over a follow up of at least four years.

Lastly, ORION-4 (NCT03705234) is aiming to demonstrate safety and efficacy of inclisiran, a first-in-class small interfering RNA that inhibits PCSK9 synthesis and has recently been shown to reduce LDL-C levels by 50% in patients on maximally tolerated statin therapy^107^. Approximately 15,000 patients with prior ASCVD will be randomized to subcutaneous inclisiran sodium or matching placebo injections. Trial participants will be followed for the incidence of major adverse cardiovascular events over a median follow up period of five years. ORION-4 will provide important outcomes data for this latest investigational cholesterol-lowering treatment.

## Conclusion and summary

Patients with clinical ASCVD are at very high risk for future atherothrombotic events. Interventions directed at modifiable risk factors such as reducing levels of LDL-C are key features in the optimal management of these patients. Multiple lines of evidence suggest that decreases in plasma LDL-C particle concentration reduce the risk of major adverse cardiac events proportional to reduction in LDL-C and the cumulative duration of exposure. For this reason, guidelines strongly support the use of LDL-C lowering therapy for secondary prevention in all patients.

Though lower LDL-C is strongly associated with lower risk for major adverse cardiac events, appropriate use of statins, ezetimibe, bempedoic acid, and PCSK9 inhibitors in at-risk populations is important for the most cost-effective utilization of these drugs. In general, all patients with a prior ASCVD event should begin treatment with maximally tolerated statin therapy. For high risk patients who remain above goal LDL-C levels on statin monotherapy, ezetimibe, bempedoic acid, and PCSK9 inhibitors should be strongly considered given data suggesting clinical benefit and no adverse safety outcomes from targeting LDL-C levels as low as 0.5 mmol/L (20 mg/dL). Given the continued evidence supporting the “lower is better” hypothesis for goal LDL-C levels, it is expected that guidelines will continue to support lower LDL-C targets in the future.

## Disclosures

PNP has no disclosures. RPG has received grants from Amgen, Anthos Therapeutics, and Daiichi Sankyo; honoraria for CME lectures from Amgen, Daiichi Sankyo, and Merck; consultant fees from Amgen, American College of Cardiology, Astra Zeneca, Boehringer-Ingelheim, Bristol-Myers-Squibb, CryoLife, CVS Caremark, Daiichi Sankyo, Eli Lilly and Company, Esperion, Gilead, GlaxoSmithKline, Janssen, Lexicon, Merck, Pfizer, St. Lukes, SAJA Pharmaceuticals, Samsung, and Servier; and institutional research grants to the TIMI Study Group at Brigham and Women’s Hospital for research he is not directly involved in from Abbott, Amgen, Aralez, AstraZeneca, Bayer HealthCare Pharmaceuticals, Inc., BRAHMS, Daiichi Sankyo, Eisai, GlaxoSmithKline, Intarcia, Janssen, MedImmune, Merck, Novartis, Pfizer, Poxel, Quark Pharmaceuticals, Roche, Takeda, The Medicines Company, and Zora Biosciences.

## Acknowledgments

We would like to thank Christopher P. Cannon, M.D. for his independent and critical review of this manuscript.
